# Transient Inhibition of mTORC1 Signaling Ameliorates Irradiation-Induced Liver Damage

**DOI:** 10.3389/fphys.2019.00228

**Published:** 2019-03-20

**Authors:** Wuping Yang, Lijian Shao, Sihong Zhu, Huan Li, Xinxin Zhang, Congcong Ding, Xincheng Wu, Rui Xu, Mengzhen Yue, Jiahui Tang, Bohai Kuang, Guangqin Fan, Qingxian Zhu, Huihong Zeng

**Affiliations:** ^1^ Medical College of Nanchang University, Nanchang, China; ^2^ Jiangxi Health Vocational College, Nanchang, China; ^3^ The Second Affiliated Hospital of Nanchang University, Nanchang, China

**Keywords:** irradiation, liver damage, mTORC1, rapamycin, apoptosis

## Abstract

Recurrent liver cancer after surgery is often treated with radiotherapy, which induces liver damage. It has been documented that activation of the TGF-β and NF-κB signaling pathways plays important roles in irradiation-induced liver pathologies. However, the significance of mTOR signaling remains undefined after irradiation exposure. In the present study, we investigated the effects of inhibiting mTORC1 signaling on irradiated livers. Male C57BL/6J mice were acutely exposed to 8.0 Gy of X-ray total body irradiation and subsequently treated with rapamycin. The effects of rapamycin treatment on irradiated livers were examined at days 1, 3, and 7 after exposure. The results showed that 8.0 Gy of irradiation resulted in hepatocyte edema, hemorrhage, and sinusoidal congestion along with a decrease of ALB expression. Exposure of mice to irradiation significantly activated the mTORC1 signaling pathway determined by pS6 and p-mTOR expression *via* western blot and immunostaining. Transient inhibition of mTORC1 signaling by rapamycin treatment consistently accelerated liver recovery from irradiation, which was evidenced by decreasing sinusoidal congestion and increasing ALB expression after irradiation. The protective role of rapamycin on irradiated livers might be mediated by decreasing cellular apoptosis and increasing autophagy. These data suggest that transient inhibition of mTORC1 signaling by rapamycin protects livers against irradiation-induced damage.

## Introduction

Liver cancer is one of the most common solid tumors in the world and results in a large amount of deaths (Ferlay et al., 2015). High recurrence occurs despite surgical treatment (Hsu et al., 2017). Radiotherapy is often considered for use when those patients with liver cancer have recurrence after surgery or are unfit for surgery (Ogata et al., 1963; Kalogeridi et al., 2015). Radiotherapy is an effective treatment to limit liver cancer growth and metastasis. On the other hand, exposure of normal hepatic tissues to irradiation results in collateral liver injury, which limits the effectiveness of radiotherapy on liver cancer. For example, 5–10% of patients will experience radiation-induced liver damage when they are exposed to a radiation dose up to 30 Gy. If the dose is increased to 43 Gy, 50% of patients will have liver damage. If the dose is further increased to 60 Gy, the most effective dose for killing liver cancer, 76% of patients will die due to hepatic failure induced by irradiation (Ingold et al., 1965; Emami et al., 1991; Dawson et al., 2001). Thus far, effective treatment of radiation-induced liver damage is unavailable, which leads to irreversible liver failure and patient death.

It has been documented that various strategies could protect against irradiation-induced liver damage. Treatment with granulocyte-colony stimulating factor (G-CSF) could prevent irradiation-induced liver damage by decreasing collagen deposition, hepatic hydroxyproline levels, and serum TGF-β1 level (Li et al., 2010). However, G-CSF treatment might lead to the exhaustion of adult stem cells (such as hematopoietic stem cells) after irradiation (Li et al., 2015). Zhang et al. reported that hepatocyte growth factor (HGF) protected rat liver cells from irradiation-induced injury through the promotion of hepatocyte regeneration (Zhang et al., 2014). Delivery of HGF-overexpressing adipose-derived mesenchymal stem cells efficiently inhibited irradiation-induced increase of liver ALT and AST (Zhang et al., 2014). Inhibition of tumor necrosis factor receptor 1 also protected against irradiation-induced liver damage (Huang et al., 2006). An antithrombotic agent—ursodeoxycholic acid—has been reported to protect hepatocytes and endothelial cells by inhibiting tumor necrosis factor-alpha production and apoptosis (Seidensticker et al., 2014). A recent study demonstrated that irradiation activated the NF-κB and TGF-β1 signaling pathways, which play an important role in irradiation-induced liver fibrosis (Hisamori et al., 2008; Cheng et al., 2015). Irradiation-induced oxidative stress on liver cells played a crucial role in liver damage. Treatment with melatonin, an antioxidant reagent, significantly decreased irradiation-induced injury in rat livers (Khan et al., 2017). Therefore, multiple factors are involved in irradiation-induced liver damage.

Mammalian target of rapamycin (mTOR) signaling integrates multiple upstream pathways, such as insulin, growth factors, cellular nutrient, energy levels and so on (Laplante and Sabatini, 2012). It plays a vital role in cell growth, proliferation, survival, protein synthesis, and autophagy (Saxton and Sabatini, 2017). mTOR interacts with other proteins to form two distinct complexes: mTOR complex 1 (mTORC1) and mTOR complex 2. mTORC1 can be activated by many factors, such as growth factors. Active mTORC1 subsequently phosphorylates translational factors, for example, ribosomal protein S6 kinase (S6K), eIF4E binding protein, and autophagy regulators, which leads to an increase of protein translation and a decrease of autophagy. It has been reported that sustained activation of mTORC1 signaling results in stem cell differentiation and exhaustion, such as in hematopoietic stem cells and hair follicle stem cells (Chen et al., 2008; Castilho et al., 2009). Inhibition of mTORC1 signaling by rapamycin treatment can extend lifespans in different organisms including worms, flies, and mice (Ehninger et al., 2014; Kennedy and Lamming, 2016). Dietary restriction can maintain intestinal stem cells and extend lifespan by repressing mTORC1 activity in Paneth cells (Yilmaz et al., 2012).

To investigate the role of mTOR signaling on irradiation-induced liver injury, mice were exposed to 8.0 Gy of X-ray total body irradiation and liver tissues were analyzed. Our data showed that the mTORC1 signaling pathway was highly activated in liver tissues after irradiation. Transient inhibition of mTORC1 signaling by rapamycin treatment consistently accelerated liver recovery after irradiation.

## Materials and Methods

### Animals and Irradiation

Eight-week-old male C57BL/6J mice (*n* = 50) were purchased from Hunan SLAC Laboratory Animal Co., Ltd (Certificate Number: SYXK 2011–0003) and shipped to Nanchang University. After a 1-week acclimation period, mice received total-body X-ray irradiation (8.0 Gy, 2.28 Gy/min, Elekta Precise accelerators). Sham-treated animals underwent the same procedures as the irradiated groups but received no irradiation (*n* = 5 mice per group). The mice were housed under a constant 12-h light: dark cycle. Food and water were provided *ad libitum*. Animals were analyzed at days 1, 3, and 7 after irradiation (*n* = 5 mice per time point). All procedures were approved by the Institutional Animal Care and Use Committee at Nanchang University.

### Rapamycin Treatment and Survival Curve

Rapamycin (>98% purity) was purchased from Dalian Meilun Biotechnology Co. Ltd. (Cas:53123-88-9; China). Rapamycin was dissolved in ethanol at 10 mg/ml and diluted in 5% Tween-80 and 5% polyethylene glycol 400 (Solarbio Science and Technology Co., Ltd., China). For rapamycin treatment, 6 h after irradiation, mice were treated with rapamycin (4 mg/kg) by subcutaneous injection and then the injection was repeated every other day until day 7 after irradiation. Mice were monitored daily until day 30 after irradiation. As a control, mice were irradiated or non-irradiated and received subcutaneous injection of the same volume of vehicle (200 μl).

### Histopathological and Immunohistochemical Examination

The hepatic tissues were fixed in 4% paraformaldehyde and then embedded in paraffin and cut into 5.0-μm-thick sections. Sections were used to perform hematoxylin and eosin (HE) staining for histological examination and to measure expression of phosphorylated S6 and Gr-1 by immunohistochemistry. Briefly, after dewaxing, dehydration, rehydration and antigen repair with microwave, paraffin sections were blocked with 3% H_2_O_2_ deionized water and subsequently incubated with the specific primary antibody against phosphorylated S6 (1:600, cell signaling technology, USA) or Gr-1 (1:1,000, Biolegend, USA) at 4°C overnight, followed by staining with horseradish peroxidase-conjugated secondary antibody. The substrate diaminobenzidine (DAB) was used for coloration. Immunostained sections were counterstained with hematoxylin to visualize the nuclei and examined under an optical microscope (Olympus, Japan).

To analyze the integrated optical density (IOD) of pS6, five visual fields (per immunohistochemical slice) were randomly selected under high magnification (10 × 40) and photographed. The IOD of pS6 in livers was calculated *via* the HMIAS-2000 image analysis system with high-resolution and multicolor imaging.

### The Levels of ALT and AST

To measure the levels of serum alanine transaminase (ALT) and aspartate transaminase (AST), serum from different groups was collected. The kits from Roche Diagnostics GmbH were used to measure levels of ALT and AST according to the manufacturer’s instructions.

### TUNEL Assay

To detect the fragmented nuclear DNA associated with apoptosis, a standard terminal deoxynucleotidyl transferase (TdT)-mediated deoxyuridine triphosphate (dUTP)-biotin nick-end labeling (TUNEL) method was employed on paraffin sections. For this purpose, the *in situ* cell apoptosis detection kit I, POD (Boster, China) was used according to the manufacturer’s instructions. Briefly, hepatic tissues were fixed in 4% paraformaldehyde and embedded with paraffin. After standard deparaffinization, hydration, incubation with 3% hydrogen peroxide at room temperature for 10 min and proteinase K at 37°C for 10 min, tissue sections were incubated: (1) with labeling buffer, TdT and DIG-dUTP (19:1:1) at 37°C for 2 h; (2) with blocking reagent at room temperature for 30 min; (3) with biotin anti-digoxin antibody at 37°C for 30 min; and (4) with SABC at 37°C for 30 min. Diaminobenzidine was used as the chromogen. For physiological positive controls, sections of mouse small intestine were subjected to the same procedure. For negative controls, some slides were incubated with label solution that did not contain TdT. The number of TUNEL-positive cells was counted from five randomly selected fields at 400× magnification per liver sample.

### Western Blot

For western blotting, the liver tissues post-irradiation were frozen in liquid nitrogen until further use. Protein extraction was carried out using the RIPA buffer (Applygen, Beijing, China). A BCA Protein Assay Kit (Applygen, Beijing, China) was used to quantitate total protein levels. Protein (40 μg per lane) was separated by SDS-PAGE. All proteins were separated on 10% gel. Proteins were transblotted to PVDF membranes (ELL) in standard Tris-glycine transfer buffer, pH 8.3, containing 0.1% SDS. After transfer, membranes were blocked for 3 h at room temperature in TBST (10 mmol/L Tris–HCl, pH 8.0, 150 mmol/L NaCl, 0.1% Tween-20) containing 5% non-fat milk powder or containing 5% BSA and incubated overnight at 4°C with anti-S6 (1:1,000, cell signaling technology, USA), anti-phospho-S6 (1:2,000, cell signaling technology, USA), anti-mTOR (1:1,000, cell signaling technology, USA), anti-phospho-mTOR (1:1,000, cell signaling technology, USA), anti-ALB (1:2,000, Affinity, USA), anti-AFP (1:2,000, Affinity, USA), anti-RIPK1 (1:3,000, Affinity, USA), anti-LC3 (1:1,000, cell signaling technology, USA), and anti-p62 (1:10,000, Abcam, UK) diluted in TBST containing 5% non-fat milk powder or 5% BSA. Membranes were then washed in TBST for 30 min, incubated with horseradish-peroxidase-conjugated goat anti-rabbit IgG, diluted 1:10,000 (Beijing Zhongshan, China) in TBST containing 5% non-fat milk powder or 5% BSA, washed in TBST for 30 min, and resolved by chemiluminescence (Beijing TIANDZ, China). All membranes were stripped and re-probed with anti-GAPDH antibody (Proteintech, China) as a loading control. The band densities from western blot were quantitated using ImageJ software^1^ and calculated according to the GAPDH band density.

### Statistical Analysis

All parameters were expressed as the mean ± standard deviation. The data were analyzed by analysis of variance (ANOVA). Differences among group means were analyzed by Student-Newman-Keuls multiple comparisons testing after one- or two-way ANOVA. A survival curve was constructed using the Kaplan-Meier method and compared using the Mantel-Cox test. Differences were considered significant at *p* < 0.05. All analyses were done with GraphPad Prism from GraphPad Software.

## Results

### Irradiation-Induced Liver Damage

**Figure 1:**
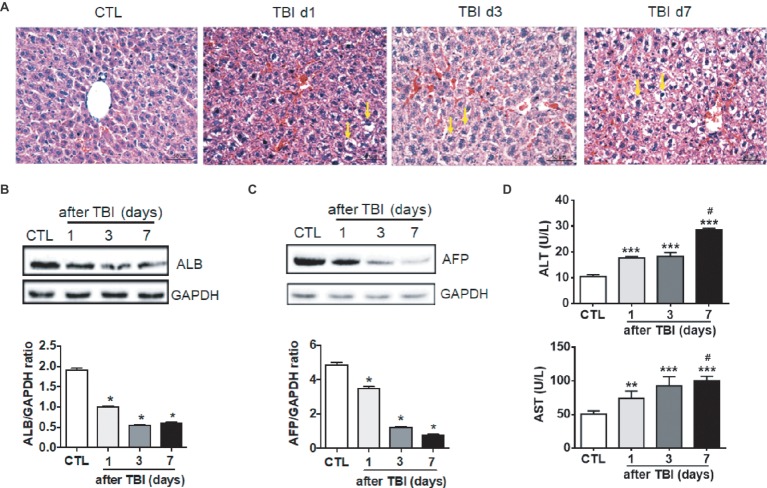
Irradiation-induced liver damage in mice. C57BL/6J mice were exposed to 8.0 Gy of total body irradiation (TBI). Liver tissues were harvested at days 1, 3, and 7 post-exposure (*n* = 6). **(A)** Representative pictures by HE staining are shown. Scale bar = 50 μm. Arrows indicate edema hepatocytes. **(B** and **C)** ALB and AFP expression in liver tissues (*n* = 3). Expression of ALB **(B)** and AFP **(C)** was detected by western blotting. GAPDH was used as a housekeeping control. Non-irradiated liver tissues (CTL) were used as controls. Expression of ALB **(B)** and AFP **(C)** was quantitated by ImageJ software. **(D)** Levels of ALT and AST in serum from different groups (*n* = 6). *p < 0.05 vs. CTL.

To investigate whether a moderate dose of X-ray irradiation induces liver damage, 8-week-old mice were acutely exposed to 8.0 Gy of total body irradiation (TBI). Morphological changes of livers were microscopically evaluated at days 1, 3, and 7 after TBI. As shown in Figure 1A, there was a normal morphology in liver tissues with intact parenchyma and sinusoids in non-irradiated mice. However, abnormal morphology including hepatocyte edema, hemorrhage, and sinusoidal congestion appeared at different time points post-irradiation. Irradiation induced not only morphological changes but also functional defects in livers. Expression of albumin (ALB) in hepatocytes was significantly decreased (up to 50%) at day 1 after irradiation when compared to normal controls. The nadir of ALB expression was reached at day 3 post-irradiation. Expression of ALB in irradiated livers persisted at low levels at day 7 after TBI (Figure 1B). The changes of alpha-fetoprotein (AFP) expression were consistently similar to those of ALB expression in livers post-irradiation (Figure 1C). The levels of serum alanine transaminase (ALT) and aspartate transaminase (AST) were increased in a time-dependent manner after irradiation (Figure 1D). These data suggested that acute exposure of livers to irradiation resulted in liver injury.

**Figure 2:**
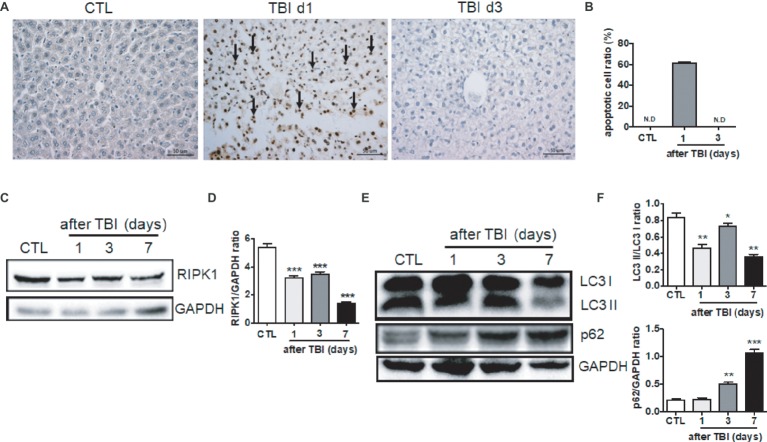
Irradiation-induced cellular apoptosis in liver tissues. **(A** and **B)** Irradiated liver tissues were collected at days 1 and 3 after TBI. Representative pictures by TUNEL staining are shown **(A**, *n* = 6**)**. Scale bar = 50 μm. Arrows indicate TUNEL-positive cells. The ratios of apoptotic cells in each field are presented in **(B)** (N.D: non-detected). **(C** and **D)** RIPK1 expression in liver tissues (*n* = 3). Expression of RIPK1 in liver tissues was detected by western blotting **(C)**. GAPDH was used as a housekeeping control. Expression of RIPK1 was quantitated by ImageJ software **(D)**. **(E** and **F)** LC3 I, LC3II, and p62 expression in liver tissues (*n* = 3). Expression of LC3 I, LC3II, and p62 **(E)** was detected by western blotting at days 1, 3, and 7 after irradiation. GAPDH was used as a housekeeping control. Expression of LC3 I, LC3II, and p62 **(F)** was quantitated by ImageJ software. The ratio of LC3 II/LC3 I was presented. *p < 0.05, **p < 0.01, ***p < 0.001 vs. CTL.

To further explore the molecular mechanisms by which irradiation induces liver damage, we measured the levels of cellular apoptosis, necroptosis, and autophagy in irradiated livers. Rare apoptotic hepatocytes were detected in non-irradiated livers while high percentages (up to 60%) of hepatocytes underwent apoptosis at day 1 after irradiation (Figures 2A,B). Those apoptotic hepatocytes were cleaned out at day 3 after irradiation. Inflammatory cell infiltration, such as granulocytes, scarcely appeared in the hepatic portal and sinusoid area at day 1 and 3 post-exposure, while a modest increase of granulocytes in the sinusoid area was detected at day 7 after irradiation (Supplementary Figure 1A).

Cellular necroptosis is a form of programmed cell death. Receptor-interacting serine/threonine-protein kinase 1 (RIPK1) plays a critical role in the initiation of necroptosis (Newton, 2015). Therefore, we measured the expression of RIPK1 protein post-irradiation. As shown in Figures 2C,D, RIPK1 expression was significantly decreased starting at days 1 and 3 after irradiation and further downregulated at day 7 when compared to non-irradiated controls. It is well known that autophagy plays a crucial role in the degradation and recycling of cellular components (Grumati and Dikic, 2018). We also evaluated the liver autophagy status after irradiation. The ratio of LC3 II/LC3 I expression in liver tissues was decreased at different time points after irradiation. The expression of p62 in liver tissues was increased starting at day 3 after irradiation (Figures 2E,F). Collectively, cellular apoptosis, necroptosis, and autophagy are involved in the acute liver damage that occurs under irradiation stress.

### Irradiation-Induced Activation of mTORC1 Signaling in Livers

**Figure 3:**
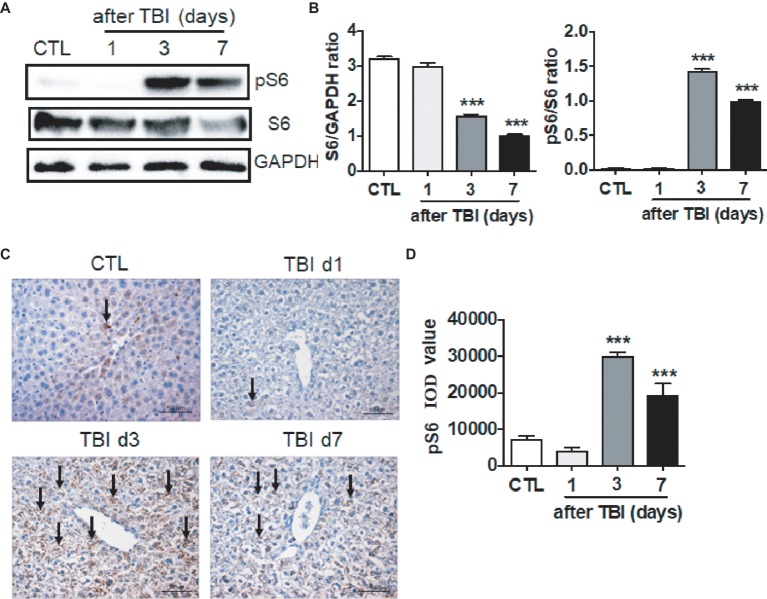
mTORC1 signaling was activated by irradiation in liver tissues. **(A** and **B)** Expression of pS6 was upregulated post-irradiation in liver tissues. Expression of pS6 and S6 **(A)** was detected by western blotting at days 1, 3, and 7 after irradiation (*n* = 3). GAPDH was used as a housekeeping control. Expression of pS6 and S6 **(B)** was quantitated by ImageJ software. **(C** and **D)** pS6 immunostaining in liver tissues (*n* = 6). Liver tissues were fixed in 4% paraformaldehyde after irradiation and pS6 immunostaining was performed as indicated in materials and methods **(C)**. Scale bar = 50 μm. Arrows indicate pS6 positive cells. Imaging software was used to analyze the integrated optical density (IOD) of pS6 (D). ***p < 0.001 vs. CTL.

It has been documented that multiple pathways are involved in irradiation-induced pathologies, for example the NF-κB and TGF-β signaling pathways and so forth (Cheng et al., 2015). The mTORC1 signaling pathway functions in nutrition/energy sensing and protein translation (Laplante and Sabatini, 2012). It is unknown whether the mTORC1 signaling pathway is activated upon irradiation. We thus harvested liver tissues at days 1, 3, and 7 after exposure, and ribosomal protein S6 and phosphorylated S6 (pS6) were examined by western blot and immunostaining. As shown in Figures 3A,B, the levels of total S6 expression were decreased at days 3 and 7 after irradiation. In physiological condition, a weak expression of pS6 was detected by western blot assay. Protein S6 in liver was highly phosphorylated starting at day 3 after irradiation when compared to non-irradiated controls. The high level of pS6 expression extended to day 7 after irradiation (Figures 3A,B). To further confirm the increased pS6 expression induced by irradiation, fixed liver tissues were used to conduct pS6 immunostaining. The increased intensity of pS6 in irradiated livers was observed at days 3 and 7 after irradiation when compared to non-irradiated controls (Figures 3C,D). Although we showed that the mTORC1 signaling pathway was activated by irradiation in liver, the functional significance of mTORC1 signaling remains obscure in liver recovery after radiation exposure.

### Inhibition of mTORC1 Signaling Ameliorates Irradiation-Induced Liver Damage

**Figure 4:**
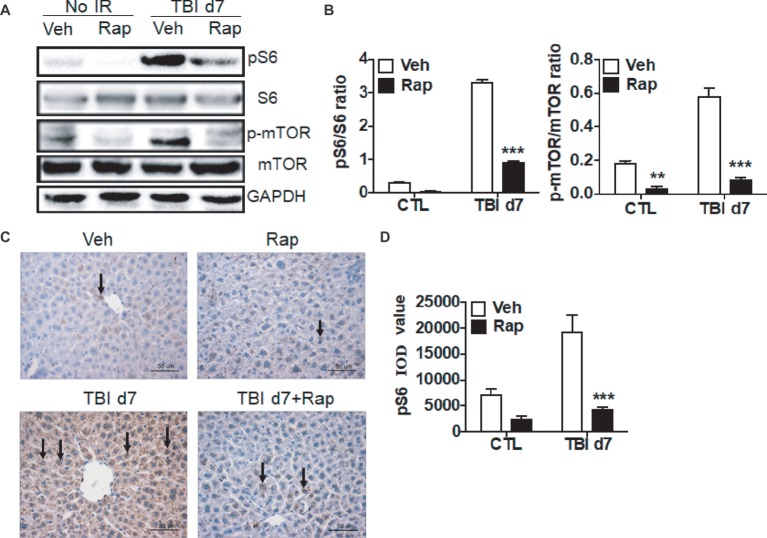
Rapamycin treatment reduced the increase of pS6 expression in irradiated liver tissues. **(A** and **B)** Increased expression of pS6 and p-mTOR by irradiation was reduced through rapamycin (Rap) treatment. Expression of pS6, S6, p-mTOR, and mTOR **(A)** was detected by western blotting at day 7 after irradiation (*n* = 3). GAPDH was used as a housekeeping control. Expression of pS6, S6, p-mTOR, and mTOR **(B)** was quantitated by ImageJ software. The ratios of pS6/S6 and p-mTOR/mTOR are presented. **(C** and **D)** pS6 immunostaining in liver tissues (*n* = 6). Liver tissues with or without rapamycin treatment were harvested at day 7 post-irradiation and fixed in 4% paraformaldehyde after irradiation, and pS6 immunostaining was performed as indicated in materials and methods **(C)**. Scale bar = 50 μm. Arrows indicate pS6-positive cells. Imaging software was used to analyze the integrated optical density (IOD) of pS6 **(D)**. **p < 0.01, ***p < 0.001 vs. Veh.

Due to the activation of the mTORC1 signaling pathway induced by irradiation as shown in Figure 3, we utilized rapamycin, a mTOR complex 1 inhibitor, to investigate its effects on the liver’s functional recovery upon total body irradiation. As shown in Figures 4A,B, rapamycin treatment decreased the expression of pS6 in non-irradiated mice. Compared to vehicle-treated controls post-exposure, the increased pS6 expression was significantly decreased in rapamycin-treated mice at day 7 after irradiation (Figures 4A,B). Expression of p-mTOR was increased in irradiated livers when compared to non-irradiated controls. Rapamycin treatment reduced the increase of p-mTOR expression in irradiated livers (Figures 4A,B). Immunostaining confirmed that rapamycin treatment efficiently blocked the increase of pS6 expression induced by irradiation (Figures 4C,D).

**Figure 5:**
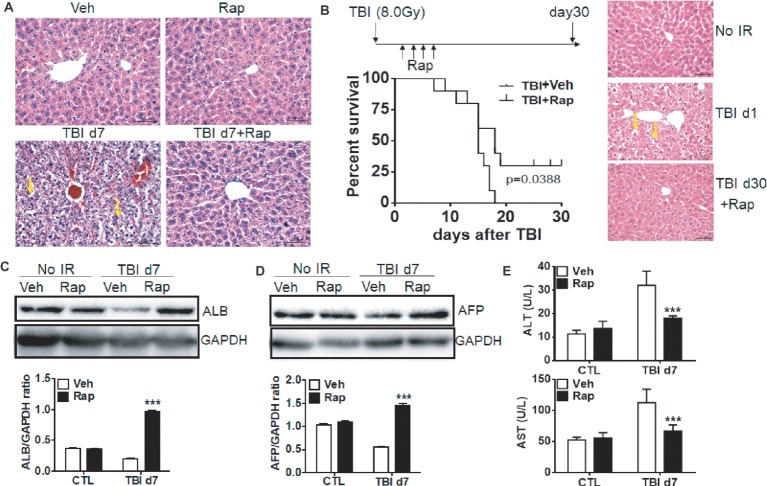
Rapamycin treatment attenuated the liver functional defect induced by irradiation. C57BL/6J mice were treated with vehicle (Veh) or rapamycin (Rap) starting at 6 h after irradiation. Liver tissues were harvested at day 7 post-exposure. **(A)** Representative pictures by HE staining are shown (*n* = 6). Scale bar = 50 μm. Arrows indicate edema hepatocytes. **(B)** Kaplan-Meier plot indicating survival of mice with vehicle or rapamycin treatment after irradiation. Irradiated mice were treated with vehicle or rapamycin as above. Mice were monitored daily until day 30 after irradiation (*n* = 16). Significance was determined by Mantel-Cox test (left panel, *p* = 0.0388). HE staining was performed in liver tissues before irradiation and day 1 and 30 after irradiation with rapamycin treatment (right panel). Scale bar = 50 μm. Arrows indicate edema hepatocytes. **(C** and **D)** Expression of ALB and AFP in liver tissues (*n* = 3). Expression of ALB **(C)** and AFP **(D)** was detected by western blotting at day 7 after irradiation. GAPDH was used as a housekeeping control. Expression of ALB **(C)** and AFP **(D)** was quantitated by ImageJ software. The ratios of ALB/GAPDH and AFP/GAPDH were presented. **(E)** Levels of ALT and AST in serum from different groups (*n* = 6). ***p < 0.001 vs. Veh.

To elucidate whether rapamycin treatment benefits liver recovery after irradiation, the changes of liver morphology were examined. Morphologically, rapamycin did not affect livers in non-irradiated mice (Figure 5A). Consistent with Figure 1, exposure of mice to irradiation induced hepatocyte edema, hemorrhage and sinusoidal congestion at day 7 post-exposure. Irradiated mice with rapamycin treatment had minor hepatocyte edema, hemorrhage and sinusoidal congestion (Figure 5A). To assess the radioprotective role of rapamycin, irradiated mice were treated with vehicle or rapamycin starting at 6 hr post-exposure. Mice were then treated with vehicle or rapamycin every other day until day 7 after irradiation and monitored daily until day 30 post-exposure. All irradiated mice with vehicle treatment died before day 17 post-exposure while 25% of irradiated mice with rapamycin treatment survived until day 30 after irradiation (Figure 5B, left panel). The morphology in rapamycin-treated livers at day 30 after irradiation was comparable to that in normal livers (Figure 5B, right panel).

These morphological changes under rapamycin treatment are consistent with functional data as shown in Figures 5C,D. Rapamycin treatment did not affect the expression of ALB and AFP in physiological condition. However, rapamycin treatment significantly ameliorated the reduction of ALB and AFP expression induced by irradiation (Figures 5C,D). The increased levels of serum ALT and AST in irradiated mice were significantly decreased upon rapamycin treatment (Figure 5E). These results display that rapamycin treatment ameliorates irradiation-induced liver injury *via* inhibition of the mTORC1 signaling pathway.

### Rapamycin Treatment Decreased Irradiation-Induced Apoptosis in Livers

**Figure 6:**
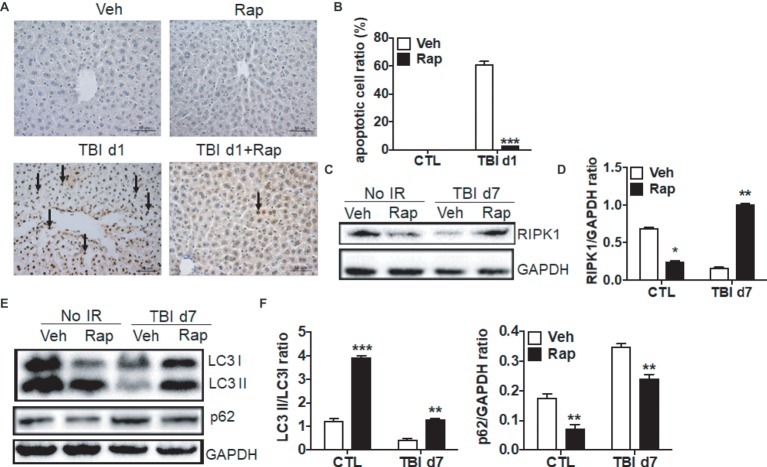
Rapamycin treatment decreased cellular apoptosis induced by irradiation. C57BL/6J mice were treated with vehicle (Veh) or rapamycin (Rap) starting at 6 h post-exposure. Liver tissues were harvested at day 1 post-exposure. **(A** and **B)** Cellular apoptosis induced by irradiation (*n* = 6). Representative pictures by TUNEL staining are shown **(A)**. Scale bar = 50 μm. Arrows indicate TUNEL-positive cells. The ratios of apoptotic cells in each field were counted and are presented in **B**. **(C** and **D)** RIPK1 expression in liver tissues (*n* = 3). Expression of RIPK1 **(C)** was detected by western blotting at day 7 after irradiation. GAPDH was used as a housekeeping control. Expression of RIPK1 **(D)** was quantitated by ImageJ software. **(E** and **F)** Expression of LC3 I, LC3 II, and p62 in liver tissues (*n* = 3). Expression of LC3 I, LC3II, and p62 **(E)** was detected by western blotting at day 7 after irradiation. GAPDH was used as a housekeeping control. Expression of LC3 I, LC3 II, and p62 **(F)** was quantitated by ImageJ software. The ratios of LC3 II/LC3 I and p62/GAPDH are presented. *p < 0.05, **p < 0.01, ***p < 0.001 vs. Veh.

To further assess the mechanism by which rapamycin treatment protects livers from irradiation, cellular apoptosis, necroptosis, and autophagy were evaluated. As shown in Figures 6A,B, rapamycin treatment did not increase the ratio of apoptotic cells in non-irradiated livers. However, rapamycin treatment significantly decreased the induction of cellular apoptosis under irradiation conditions (Figures 6A,B). Granulocyte infiltration in livers at day 7 after irradiation was significantly declined under rapamycin treatment (Supplementary Figure 1B). For necroptosis, rapamycin treatment decreased the expression of RIPK1 in physiological condition (Figures 6C,D). The decreased expression of RIPK1 induced by irradiation was recovered by rapamycin treatment. For autophagy, rapamycin treatment increased the ratio of LC3 II/LC3 I and decreased the expression of p62 under physiological condition. The decreased ratio of LC3 II/LC3 I and increased expression of p62 after irradiation was partially recovered by rapamycin treatment (Figures 6E,F). These data indicated that rapamycin treatment protected livers against irradiation-induced damage through multiple mechanisms.

## Discussion

Previous studies have shown that hepatocytes are more radioresistant than other cells, such as hematopoietic cells (Alati et al., 1988; Christiansen et al., 2004; Chang et al., 2017). In clinical practice, the liver is often considered to be radiosensitive. However, this observation is based on patients treated with liver irradiation when the liver was previously cirrhotic, or patients who were treated with both irradiation and chemotherapy. Furthermore, abdominal radiotherapy dosage is limited because of radiation-induced liver disease. Liver radiotherapy is clinically considered in the case of recurrence after surgery, advanced liver cancer, late stages of liver cancer, and metastasized tumors from other organs (Zhou et al., 2007; Kalogeridi et al., 2015). Moreover, the liver tissues can be targeted when radiotherapy has been applied to tumors in the abdominal and pelvic cavities (Cheng et al., 2005). Because irradiation-induced normal tissue injuries limit the application of high radiation doses on liver cancer, only 30% of patients with liver cancer can be treated with proper doses of irradiation (Kalogeridi et al., 2015). The number of patients with liver cancers increases every year, resulting in the increasing requirement of radiotherapy. Therefore, it is important to reduce and prevent the negative effects of radiotherapy on normal liver tissues.

However, no effective therapeutic approach exists to prevent irradiation-induced liver damage. Amifostine is an FDA-approved prodrug for radiation countermeasure, which was previously used for protecting normal tissues from chemotherapy- and radiotherapy-induced undesired side effects (Johnke et al., 2014). However, it was rarely used in clinic due to patients’ discomfort of intravenous administration and its high toxicity. It has been reported that treatment with G-CSF (filgrastim), another FDA-approved radiation medical countermeasure, attenuated radiation-induced hematopoietic syndrome of acute radiation syndrome (Pospisil et al., 1999; Moroni et al., 2013). In an irradiation-induced liver damage model, G-CSF treatment decreased irradiation-induced collagen deposition in livers and serum TGF-β1 level (Li et al., 2010). However, a recent report showed that G-CSF treatment accelerated the exhaustion of hematopoietic stem cells after irradiation (Li et al., 2015).

In the current study, we demonstrated that total body irradiation induced liver damage, which was proved by abnormal liver morphology and decreasing ALB expression and increasing levels of serum ALT and AST post-exposure. These pathological changes after irradiation might be attributed to the activation of the mTORC1 signaling pathway, including increased levels of pS6 and p-mTOR in irradiated livers. Total levels of S6 expression were decreased in livers after irradiation, which might be due to irradiation-induced ribosomal stress responses. Rapamycin inhibited the activation of mTORC1 and corrected irradiation-induced liver dysfunction by decreasing cellular apoptosis and serum ALT and AST levels in addition to increasing autophagy. These findings indicate that rapamycin treatment attenuates irradiation-induced acute liver damage to protect mice from death. However, it is unknown whether there are long-term effects of rapamycin treatment on irradiation-induced liver damage, which will be explored in our future studies. In addition, we used total body irradiation to investigate the protective role of rapamycin on irradiated mice. It would be more clinically relevant if local liver irradiation can be used in the near future.

Irradiation-induced hepatic injury might be attributed to the infiltration of inflammatory cells in the irradiated livers, although irradiation also triggers significant damage to vessels in livers along with the reduction of blood supply. This phenomenon was extensively investigated by Dr. Ramadori’s group (Malik et al., 2010; Sultan et al., 2013). Many granulocytes, labeled with Gr-1, CD11b/c, and MPO, were detected and specifically accumulated in the portal area at 3 and 6 hr after rat livers were locally irradiated with a single dose of 25 Gy γ-irradiation. Acute granulocyte recruitment in the portal area after irradiation might be mediated by (myo)fibroblast-derived chemokines, such as CCL2, CXCL2, CXCR2, and so forth (Malik et al., 2010; Sultan et al., 2013). Single-dose irradiation-induced granulocyte infiltration in the hepatic portal area was significantly decreased at 12 and 24 h post-exposure in rats (Malik et al., 2010; Sultan et al., 2013). This is consistent with our current observation, showing that rare granulocytes were recognized in both the portal and sinusoid areas at days 1 and 3 after 8 Gy of total-body X-ray irradiation in mice. However, we observed a slight increase of granulocytes in the sinusoid area at day 7 after irradiation, which was attenuated by rapamycin treatment. The increased distribution of granulocytes in livers after 8 Gy irradiation was also supported by a fractionated irradiation study (Rave-Frank et al., 2013). It showed that increased numbers of granulocytes were distributed in hepatic parenchyma at 3 months after rat livers were exposed to 2 Gy/day, with a total dose of 60 Gy γ-irradiation. Given that rapamycin has an ability to limit inflammation (Wang et al., 2014; De Luna-Preitschopf et al., 2017), rapamycin treatment starting at 6 h post-irradiation might thus attenuate chemokine production and granulocyte infiltration to ameliorate radiation-induced liver damage.

Another cause of irradiation-induced hepatic injury is the induction of cellular apoptosis in livers, which can also result from irradiation-induced DNA damage. We therefore demonstrated that cellular apoptosis was induced by irradiation in liver tissues using TUNEL staining. Our data indicated that cellular apoptosis was markedly increased at day 1 after irradiation. Subsequent results showed that rapamycin treatment significantly attenuated the cellular apoptosis induced by irradiation in liver tissues. Cellular necroptosis is another type of cell death, which plays a crucial role in TNF-induced cell death. RIPK1 and RIPK3 are core components of TNF-induced signaling complexes involved in necroptosis and apoptosis (Moriwaki and Chan, 2013; Menon et al., 2017). Deletion of RIPK1 in intestinal epithelial cells causes severe intestinal inflammation to develop, mediated by cellular apoptosis. Deficiency of Caspase 8 or FADD decreases intestinal cell apoptosis in RIPK1 deleted cells, while epithelial cells appear to exhibit cellular necroptosis in double deficiency of RIPK1 and FADD. Deletion of RIPK1 and FADD-induced necroptosis can be rescued by RIPK3 mutation (Takahashi et al., 2014). Keratinocyte-specific deletion of RIPK1 results in cellular apoptosis and necroptosis along with skin inflammation, which could be rescued by RIPK3 deficiency (Dannappel et al., 2014). Our current data showed that RIPK1 expression was decreased in liver tissues after irradiation, which might accelerate RIPK3-mediated necroptosis post-exposure. Rapamycin treatment can significantly increase the expression of RIPK1 in irradiated liver tissues, which might block RIPK3-mediated necroptosis and protect liver tissues from irradiation-induced damage. These assumptions about the roles of RIPK1 and RIPK3 under irradiation will be further tested in our future studies.

Autophagy is a dynamic process in which the cells digest and recycle their own cytoplasmic elements. It maintains healthy cellular homeostasis by degrading damaged and useless cellular components. It is well known that many genes, including the autophagy-related (ATG) genes LC3 and p62, participate in the autophagy process. It has been reported that p62 is required for the aggregation of ubiquitinylated proteins and plays important roles in autophagic clearance (Komatsu et al., 2007). Many signaling pathways regulate the autophagy process, such as mTOR and PI3K/Beclin-1 signaling. Irradiation-induced autophagy has been reported in several different types of tumors and can function as a pro-survival and pro-death process (Kim et al., 2009; Dalby et al., 2010). Our data showed that the ratio of LC3 II/LC3 I expression was decreased and expression of p62 protein was increased in liver tissues after irradiation, which indicates that the autophagy process was transiently inhibited by irradiation. Rapamycin treatment inhibited the mTORC1 signaling pathway induced by irradiation and activated the autophagy process to clear damaged cells. The ratio of LC3 II/LC3 I expression was increased, and expression of p62 protein was decreased in irradiated liver tissues under rapamycin treatment. We currently provided evidence showing that treatment with rapamycin promoted necroptosis and autophagic programming in irradiated livers. However, we did not have direct evidence with cellular necroptosis and autophagy using transmission electron microscopy.

On the other hand, it has been documented that inhibiting mTORC1 signaling on cancer cells resulted in enhancing irradiation-induced cytotoxicity to suppress tumor growth (Dumont and Bischoff, 2012; Miyahara et al., 2017). The effects of rapamycin on cancer cells are mainly mediated by autophagic cell death, inhibiting DNA damage repair and limiting tumor angiogenesis and so on (Dumont and Bischoff, 2012; Li et al., 2016). In the present study, we mainly investigated the effects of rapamycin on irradiated normal tissues. Our result showed that transient rapamycin treatment did not affect liver morphology and function in physiological condition, while it indeed protected irradiation-induced liver damage by decreasing apoptosis and necroptosis and increasing cellular autophagy. However, it is unknown why the different responses of rapamycin exist in normal and tumor tissues. Radioprotection of rapamycin on normal tissues is also supported by a recent study, showing that rapamycin treatment prevented irradiation-induced salivary hypofunction in swine and human submandibular gland HSG cells (Zhu et al., 2016). Recent data from our lab have shown that rapamycin treatment after irradiation protected kidneys in mice by inhibiting cellular apoptosis (Shao et al., 2018). However, the effects of rapamycin treatment on liver and kidney before irradiation remain warranted in our future investigation.

Based on our results, irradiation causes activation of the mTORC1 signaling pathway while rapamycin, an inhibitor of mTORC1, attenuates irradiation-induced damage to mouse livers. In conclusion, our results show that transient treatment with rapamycin is likely to provide significant protection against irradiation-induced liver damage. These findings hold the promise that long-term administration of rapamycin may provide protection against higher doses of irradiation, although it remains necessary to validate these findings in the near future.

## Data Availability

All datasets generated for this study are included in the manuscript and/or the Supplementary Files.

## Author Contributions

WY, LS, and SZ designed the research, performed research, analyzed data, and wrote the paper. HL, XZ, CD, XW, RX, MY, and JT performed research and analyzed data. BK, GF, and QZ performed research. HZ designed research, analyzed data, and wrote the paper.

### Conflict of Interest Statement

The authors declare that the research was conducted in the absence of any commercial or financial relationships that could be construed as a potential conflict of interest.

## Footnotes

^1^http://rsbweb.nih.gov/ij

## Supplementary Material

SUPPLEMENTARY FIGURE 1 Rapamycin treatment decreased numbers of granulocytes in irradiated livers. **(A)** Gr-1 immunostaining in livers at days 1, 3 and 7 after irradiation (upper panel, n = 6). Numbers of Gr-1+ cells were counted and presented as numbers (No.) of Gr-1+ cells in each field (lower panel, 400× magnification). **(B)** Gr-1 immunostaining was performed at day 7 after the treatment (upper panel, n = 6). Changes of numbers of Gr-1+ cells in each field were presented after irradiation and/or rapamycin treatment (lower panel, 400× magnification). ***p < 0.001 vs non-irradiated control (CTL). #p < 0.01 vs. irradiated mice with rapamycin treatment.

## References

[ref1] Alati T. Van Cleeff M. Strom S. C. Jirtle R. L. (1988). Radiation sensitivity of adult human parenchymal hepatocytes. Radiat. Res. 115, 152–160. 10.2307/3577063, PMID: 3393629

[ref2] Castilho R. M. Squarize C. H. Chodosh L. A. Williams B. O. Gutkind J. S. (2009). mTOR mediates Wnt-induced epidermal stem cell exhaustion and aging. Cell Stem Cell 5, 279–289. 10.1016/j.stem.2009.06.017, PMID: 19733540 PMC2939833

[ref3] Chang J. Wang Y. Pathak R. Sridharan V. Jones T. Mao X. W.. (2017). Whole body proton irradiation causes acute damage to bone marrow hematopoietic progenitor and stem cells in mice. Int. J. Radiat. Biol. 93, 1312–1320. 10.1080/09553002.2017.1356941, PMID: 28782442 PMC6693495

[ref4] Chen C. Liu Y. Liu R. Ikenoue T. Guan K. L. Liu Y.. (2008). TSC-mTOR maintains quiescence and function of hematopoietic stem cells by repressing mitochondrial biogenesis and reactive oxygen species. J. Exp. Med. 205, 2397–2408. 10.1084/jem.20081297, PMID: 18809716 PMC2556783

[ref5] Cheng J. C. Liu H. S. Wu J. K. Chung H. W. Jan G. J. (2005). Inclusion of biological factors in parallel-architecture normal-tissue complication probability model for radiation-induced liver disease. Int. J. Radiat. Oncol. Biol. Phys. 62, 1150–1156. 10.1016/j.ijrobp.2004.12.031, PMID: 15990021

[ref6] Cheng W. Xiao L. Ainiwaer A. Wang Y. Wu G. Mao R.. (2015). Molecular responses of radiation-induced liver damage in rats. Mol. Med. Rep. 11, 2592–2600. 10.3892/mmr.2014.3051, PMID: 25483171 PMC4337597

[ref7] Christiansen H. Saile B. Neubauer-Saile K. Tippelt S. Rave-Frank M. Hermann R. M.. (2004). Irradiation leads to susceptibility of hepatocytes to TNF-alpha mediated apoptosis. Radiother. Oncol. 72, 291–296. 10.1016/j.radonc.2004.07.001, PMID: 15450727

[ref8] Dalby K. N. Tekedereli I. Lopez-Berestein G. Ozpolat B. (2010). Targeting the prodeath and prosurvival functions of autophagy as novel therapeutic strategies in cancer. Autophagy 6, 322–329. 10.4161/auto.6.3.11625 20224296 PMC2914492

[ref9] Dannappel M. Vlantis K. Kumari S. Polykratis A. Kim C. Wachsmuth L.. (2014). RIPK1 maintains epithelial homeostasis by inhibiting apoptosis and necroptosis. Nature 513, 90–94. 10.1038/nature13608, PMID: 25132550 PMC4206266

[ref10] Dawson L. A. Ten Haken R. K. Lawrence T. S. (2001). Partial irradiation of the liver. Semin. Radiat. Oncol. 11, 240–246. 10.1053/srao.2001.23485, PMID: 11447581

[ref11] De Luna-Preitschopf A. Zwickl H. Nehrer S. Hengstschlager M. Mikula M. (2017). Rapamycin maintains the chondrocytic phenotype and interferes with inflammatory cytokine induced processes. Int. J. Mol. Sci. 18:E1494. 10.3390/ijms18071494, PMID: 28696356 PMC5535984

[ref12] Dumont F. J. Bischoff P. (2012). Disrupting the mTOR signaling network as a potential strategy for the enhancement of cancer radiotherapy. Curr. Cancer Drug Targets 12, 899–924. 10.2174/156800912803251243, PMID: 22831276

[ref13] Ehninger D. Neff F. Xie K. (2014). Longevity, aging and rapamycin. Cell. Mol. Life Sci. 71, 4325–4346. 10.1007/s00018-014-1677-1, PMID: 25015322 PMC4207939

[ref14] Emami B. Lyman J. Brown A. Coia L. Goitein M. Munzenrider J. E.. (1991). Tolerance of normal tissue to therapeutic irradiation. Int. J. Radiat. Oncol. Biol. Phys. 21, 109–122. 10.1016/0360-3016(91)90171-Y, PMID: 2032882

[ref15] Ferlay J. Soerjomataram I. Dikshit R. Eser S. Mathers C. Rebelo M.. (2015). Cancer incidence and mortality worldwide: sources, methods and major patterns in GLOBOCAN 2012. Int. J. Cancer 136, E359–E386. 10.1002/ijc.29210, PMID: 25220842

[ref16] Grumati P. Dikic I. (2018). Ubiquitin signaling and autophagy. J. Biol. Chem. 293, 5404–5413. 10.1074/jbc.TM117.000117, PMID: 29187595 PMC5900779

[ref17] Hisamori S. Tabata C. Kadokawa Y. Okoshi K. Tabata R. Mori A.. (2008). All-trans-retinoic acid ameliorates carbon tetrachloride-induced liver fibrosis in mice through modulating cytokine production. Liver Int. 28, 1217–1225. 10.1111/j.1478-3231.2008.01745.x, PMID: 18397230

[ref18] Hsu H. Y. Yu M. C. Lee C. W. Tsai H. I. Sung C. M. Chen C. W. (2017). RAM score is an effective predictor for early mortality and recurrence after hepatectomy for hepatocellular carcinoma. BMC Cancer 17:742. 10.1186/s12885-017-3748-9 29121890 PMC5680811

[ref19] Huang X. W. Yang J. Dragovic A. F. Zhang H. Lawrence T. S. Zhang M. (2006). Antisense oligonucleotide inhibition of tumor necrosis factor receptor 1 protects the liver from radiation-induced apoptosis. Clin. Cancer Res. 12, 2849–2855. 10.1158/1078-0432.ccr-06-0360, PMID: 16675580

[ref20] Ingold J. A. Reed G. B. Kaplan H. S. Bagshaw M. A. (1965). Radiation hepatitis. Am. J. Roentgenol. Radium Ther. Nucl. Med. 93, 200–208. PMID: 14243011

[ref21] Johnke R. M. Sattler J. A. Allison R. R. (2014). Radioprotective agents for radiation therapy: future trends. Future Oncol. 10, 2345–2357. 10.2217/fon.14.175, PMID: 25525844

[ref22] Kalogeridi M. A. Zygogianni A. Kyrgias G. Kouvaris J. Chatziioannou S. Kelekis N.. (2015). Role of radiotherapy in the management of hepatocellular carcinoma: a systematic review. World J. Hepatol. 7, 101–112. 10.4254/wjh.v7.i1.101, PMID: 25625001 PMC4295187

[ref23] Kennedy B. K. Lamming D. W. (2016). The mechanistic target of rapamycin: the grand conductor of metabolism and aging. Cell Metab. 23, 990–1003. 10.1016/j.cmet.2016.05.009, PMID: 27304501 PMC4910876

[ref24] Khan S. Adhikari J. S. Rizvi M. A. Chaudhury N. K. (2017). Melatonin attenuates (60) Co gamma-ray-induced hematopoietic, immunological and gastrointestinal injuries in C57BL/6 male mice. Environ. Toxicol. 32, 501–518. 10.1002/tox.22254, PMID: 26948951

[ref25] Kim K. W. Moretti L. Mitchell L. R. Jung D. K. Lu B. (2009). Combined Bcl-2/mammalian target of rapamycin inhibition leads to enhanced radiosensitization via induction of apoptosis and autophagy in non-small cell lung tumor xenograft model. Clin. Cancer Res. 15, 6096–6105. 10.1158/1078-0432.ccr-09-0589, PMID: 19773376 PMC2756330

[ref26] Komatsu M. Waguri S. Koike M. Sou Y. S. Ueno T. Hara T.. (2007). Homeostatic levels of p62 control cytoplasmic inclusion body formation in autophagy-deficient mice. Cell 131, 1149–1163. 10.1016/j.cell.2007.10.035, PMID: 18083104

[ref27] Laplante M. Sabatini D. M. (2012). mTOR signaling in growth control and disease. Cell 149, 274–293. 10.1016/j.cell.2012.03.017, PMID: 22500797 PMC3331679

[ref28] Li Y. Liu F. Wang Y. Li D. Guo F. Xu L.. (2016). Rapamycin-induced autophagy sensitizes A549 cells to radiation associated with DNA damage repair inhibition. Thorac. Cancer 7, 379–386. 10.1111/1759-7714.12332, PMID: 27385978 PMC4930955

[ref29] Li C. Lu L. Zhang J. Huang S. Xing Y. Zhao M.. (2015). Granulocyte colony-stimulating factor exacerbates hematopoietic stem cell injury after irradiation. Cell Biosci. 5:65. 10.1186/s13578-015-0057-3, PMID: 26609358 PMC4659162

[ref30] Li N. Zhang L. Li H. Fang B. (2010). Administration of granulocyte colony-stimulating factor ameliorates radiation-induced hepatic fibrosis in mice. Transplant. Proc. 42, 3833–3839. 10.1016/j.transproceed.2010.09.010, PMID: 21094866

[ref31] Malik I. A. Moriconi F. Sheikh N. Naz N. Khan S. Dudas J.. (2010). Single-dose gamma-irradiation induces up-regulation of chemokine gene expression and recruitment of granulocytes into the portal area but not into other regions of rat hepatic tissue. Am. J. Pathol. 176, 1801–1815. 10.2353/ajpath.2010.090505, PMID: 20185578 PMC2843471

[ref32] Menon M. B. Gropengiesser J. Fischer J. Novikova L. Deuretzbacher A. Lafera J.. (2017). p38(MAPK)/MK2-dependent phosphorylation controls cytotoxic RIPK1 signalling in inflammation and infection. Nat. Cell Biol. 19, 1248–1259. 10.1038/ncb3614, PMID: 28920954

[ref33] Miyahara H. Yadavilli S. Natsumeda M. Rubens J. A. Rodgers L. Kambhampati M.. (2017). The dual mTOR kinase inhibitor TAK228 inhibits tumorigenicity and enhances radiosensitization in diffuse intrinsic pontine glioma. Cancer Lett. 400, 110–116. 10.1016/j.canlet.2017.04.019, PMID: 28450157 PMC5569904

[ref34] Moriwaki K. Chan F. K. (2013). RIP3: a molecular switch for necrosis and inflammation. Genes Dev. 27, 1640–1649. 10.1101/gad.223321.113, PMID: 23913919 PMC3744722

[ref35] Moroni M. Ngudiankama B. F. Christensen C. Olsen C. H. Owens R. Lombardini E. D.. (2013). The Gottingen minipig is a model of the hematopoietic acute radiation syndrome: G-colony stimulating factor stimulates hematopoiesis and enhances survival from lethal total-body gamma-irradiation. Int. J. Radiat. Oncol. Biol. Phys. 86, 986–992. 10.1016/j.ijrobp.2013.04.041, PMID: 23845847 PMC3710733

[ref36] Newton K. (2015). RIPK1 and RIPK3: critical regulators of inflammation and cell death. Trends Cell Biol. 25, 347–353. 10.1016/j.tcb.2015.01.001, PMID: 25662614

[ref37] Ogata K. Hizawa K. Yoshida M. Kitamuro T. Akagi G. Kagawa K.. (1963). Hepatic injury following irradiation—a morphologic study. Tokushima J. Exp. Med. 10, 240–251. PMID: 14049847

[ref38] Pospisil M. Hofer M. Netikova J. Hola J. Znojil V. Vacha J.. (1999). Pretreatment with granulocyte colony-stimulating factor reduces myelopoiesis in irradiated mice. Radiat. Res. 151, 363–367. 10.2307/3579949, PMID: 10073675

[ref39] Rave-Frank M. Malik I. A. Christiansen H. Naz N. Sultan S. Amanzada A.. (2013). Rat model of fractionated (2 Gy/day) 60 Gy irradiation of the liver: long-term effects. Radiat. Environ. Biophys. 52, 321–338. 10.1007/s00411-013-0468-7, PMID: 23595725

[ref40] Saxton R. A. Sabatini D. M. (2017). mTOR Signaling in growth, metabolism, and disease. Cell 169, 361–371. 10.1016/j.cell.2017.03.035, PMID: 28388417

[ref41] Seidensticker M. Seidensticker R. Damm R. Mohnike K. Pech M. Sangro B.. (2014). Prospective randomized trial of enoxaparin, pentoxifylline and ursodeoxycholic acid for prevention of radiation-induced liver toxicity. PLoS One 9:e112731. 10.1371/journal.pone.0112731, PMID: 25393877 PMC4231047

[ref42] Shao L. Yang W. Xu R. Zhu S. Huang Y. Li H. (2018). Inhibition of mTORC1 signaling protects kidney from irradiation-induced toxicity via accelerating recovery of renal stem-like cells. Stem Cell Res. Ther. 9:219. 10.1186/s13287-018-0963-5 30107854 PMC6092808

[ref43] Sultan S. Cameron S. Ahmad S. Malik I. A. Schultze F. C. Hielscher R.. (2013). Serum Lipocalin2 is a potential biomarker of liver irradiation damage. Liver Int. 33, 459–468. 10.1111/liv.12073, PMID: 23331620

[ref44] Takahashi N. Vereecke L. Bertrand M. J. Duprez L. Berger S. B. Divert T.. (2014). RIPK1 ensures intestinal homeostasis by protecting the epithelium against apoptosis. Nature 513, 95–99. 10.1038/nature13706, PMID: 25186904

[ref45] Wang W. Yan J. Wang H. Shi M. Zhang M. Yang W.. (2014). Rapamycin ameliorates inflammation and fibrosis in the early phase of cirrhotic portal hypertension in rats through inhibition of mTORC1 but not mTORC2. PLoS One 9:e83908. 10.1371/journal.pone.0083908, PMID: 24404143 PMC3880276

[ref46] Yilmaz O. H. Katajisto P. Lamming D. W. Gultekin Y. Bauer-Rowe K. E. Sengupta S.. (2012). mTORC1 in the Paneth cell niche couples intestinal stem-cell function to calorie intake. Nature 486, 490–495. 10.1038/nature11163, PMID: 22722868 PMC3387287

[ref47] Zhang J. Zhou S. Zhou Y. Feng F. Wang Q. Zhu X.. (2014). Hepatocyte growth factor gene-modified adipose-derived mesenchymal stem cells ameliorate radiation induced liver damage in a rat model. PLoS One 9:e114670. 10.1371/journal.pone.0114670, PMID: 25501583 PMC4264768

[ref48] Zhou Z. H. Liu L. M. Chen W. W. Men Z. Q. Lin J. H. Chen Z.. (2007). Combined therapy of transcatheter arterial chemoembolisation and three-dimensional conformal radiotherapy for hepatocellular carcinoma. Br. J. Radiol. 80, 194–201. 10.1259/bjr/33521596, PMID: 17038412

[ref49] Zhu Z. Pang B. Iglesias-Bartolome R. Wu X. Hu L. Zhang C.. (2016). Prevention of irradiation-induced salivary hypofunction by rapamycin in swine parotid glands. Oncotarget 7, 20271–20281. 10.18632/oncotarget.7941, PMID: 26958808 PMC4991453

