# Supplementary figures and images for: Transient Inhibition of mTORC1 Signaling Ameliorates Irradiation-Induced Liver Damage
Source: Front Physiol. 2019 Mar 20;10:228. doi: 10.3389/fphys.2019.00228 (PMC6449701; doi:10.3389/fphys.2019.00228)

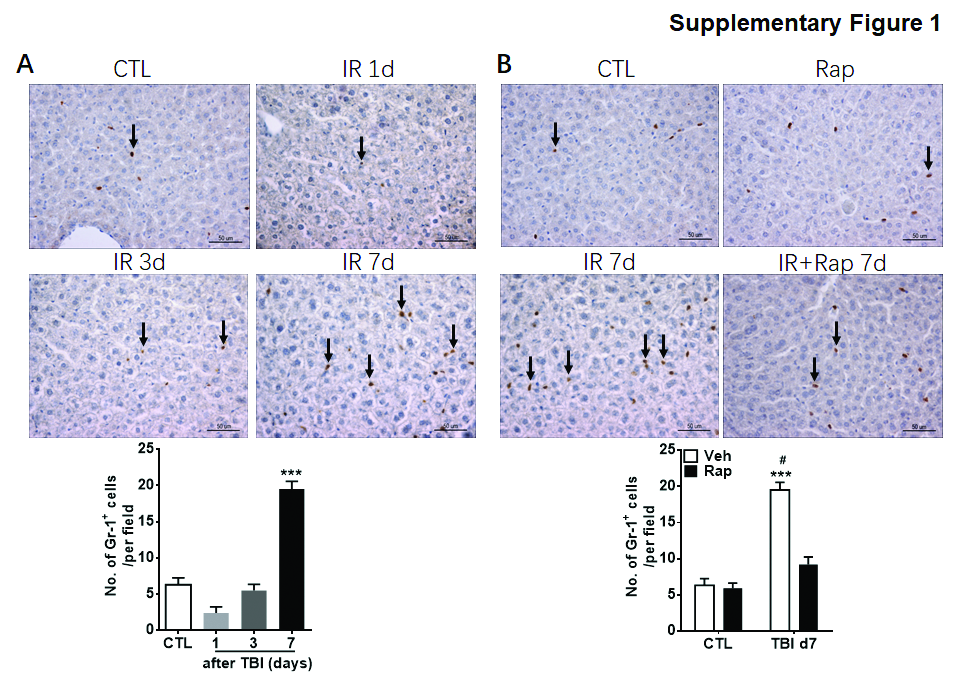

Supplement: SUPPLEMENTARY FIGURE 1 — Rapamycin treatment decreased numbers of granulocytes in irradiated livers. (A) Gr-1 immunostaining in livers at days 1, 3 and 7 after irradiation (upper panel, n = 6). Numbers of Gr-1+ cells were counted and presented as numbers (No.) of Gr-1+ cells in each field (lower panel, 400× magnification). (B) Gr-1 immunostaining was performed at day 7 after the treatment (upper panel, n = 6). Changes of numbers of Gr-1+ cells in each field were presented after irradiation and/or rapamycin treatment (lower panel, 400× magnification). ***p < 0.001 vs non-irradiated control (CTL). #p < 0.01 vs. irradiated mice with rapamycin treatment. [file Image_1.TIF]
